# Convergence and Performance Analysis of a Particle Swarm Optimization Algorithm for Optical Tuning of Gold Nanohole Arrays

**DOI:** 10.3390/ma17040807

**Published:** 2024-02-07

**Authors:** Margherita Angelini, Luca Zagaglia, Franco Marabelli, Francesco Floris

**Affiliations:** Department of Physics, University of Pavia, Via Bassi 6, 27100 Pavia, Italy; luca.zagaglia@unipv.it (L.Z.); francesco.floris@unipv.it (F.F.)

**Keywords:** FDTD simulation, particle swarm optimization algorithm, gold nanohole arrays

## Abstract

Gold nanohole arrays, hybrid metal/dielectric metasurfaces composed of periodically arranged air holes in a thick gold film, exhibit versatile support for both localized and propagating surface plasmons. Leveraging their capabilities, particularly in surface plasmon resonance-oriented applications, demands precise optical tuning. In this study, a customized particle swarm optimization algorithm, implemented in Ansys Lumerical FDTD, was employed to optically tune gold nanohole arrays treated as bidimensional gratings following the Bragg condition. Both square and triangular array dispositions were considered. Convergence and evolution of the particle swarm optimization algorithm were studied, and a mathematical model was developed to interpret its outcomes.

## 1. Introduction

Surface plasmons (SPs) are collective charge density oscillations localized at the interface between two materials having the dielectric function with the opposite sign, namely, a metal and a dielectric [1]. Due to their bound nature, they are not only extremely sensitive to refractive index changes at the interface but can also enhance different physical phenomena, such as fluorescence or Raman effect, largely exploited for biosensing applications [2,3,4,5]. To increase the sensing performances concerning the excitation of SPs, the rational design of plasmonic nanostructures has attracted much interest in recent years [6,7]. It has been shown that the SPs’ properties and thus sensing performances depend on various factors of the overall system, including both geometrical and morphological features [8]. For this reason, there is a significant interest in the optimization of such parameters allowing the design and tailoring of plasmonic nanomaterials with high performance. Uniform metal films supporting propagating SPs, namely surface plasmon polaritons (SPPs), have been the first to be used in this field, conferring sensing performance but generally requiring bulky optical systems to couple the electromagnetic radiation to SPPs [9]. For this reason, nanoparticle-based ones have been largely replacing such systems due to low fabrication costs and the easy coupling with SPs that are, in this case, localized surface plasmons (LSPRs) [9,10]. In recent years, technological development has provided new and competitive techniques for the nanofabrication of plasmonic nanostructures, such as plasmonic metasurfaces, offering the possibility of manipulating plasmonic features [1,11,12]. Among them, gold nanohole arrays (GNAs) have been widely studied due to the exhibited extraordinary optical transmission [13,14,15,16] but also for the peculiar potential to control light coupling with both SPPs and LSPRs. GNAs are hybrid metal/dielectric metasurfaces consisting of periodically arranged air holes in a thick gold layer. It has been shown that GNAs are good candidates for several biosensing applications, like surface plasmon resonance (SPR) [17,18,19,20] and plasmon-enhanced fluorescence (PEF) [4,21,22,23,24,25,26,27]. Nevertheless, the GNAs’ optical properties and thus their sensing performance are influenced by the geometrical surface parameters, namely, the array periodicity, the air hole shape, and the thickness of the gold layer [8].

For 20 years, we have been working on plasmonic-based biosensors with GNAs by using colloidal lithography [18,28,29]. The main advantage of this technique resides in its low cost and easy implementation. Nevertheless, control over the nanofabrication parameters determining the GNA geometrical features is challenging, limiting the tunability of the plasmonic response for the desired application. Since our plasmonic nanostructure has been shown to be very promising for both SPR and PEF detection-based devices, we are currently looking for an alternative nanofabrication procedure based on UV-lithography to provide reliability and tunability of the optical response together with large-scale fabrication compliance. Consequently, to achieve the desired response, a precise tailoring of all these features is needed. Therefore, Maxwell’s equations have to be rigorously solved in both time and space domains, and fortunately, it can be achieved with different types of computational methods. Moreover, it is not straightforward to manage the electromagnetic feature optimization through the evolution of the optical properties. An interesting solution is represented by evolutionary algorithms, such as the genetic algorithm and the particle swarm optimization (PSO) algorithm [30,31,32,33,34,35], or, more recently, artificial intelligence-based ones [36,37,38]. The PSO and GA are based on different philosophies: the first relies upon “social” swarm behavior, and the GA relies upon genetic encoding and natural selection [33]. In the literature, the PSO has been widely used to optimize RF antenna array properties and applied in photonics for the design and optimization of several dielectric devices, such as grating couplers [39]. Generally, due to the presence of a complex dielectric function, the simulations of plasmonic systems are computationally demanding. The intuitive mathematical structure, combined with easy parameter manipulation and a high capacity to control convergence while preventing stagnation issues—common in genetic algorithms (GAs)—has positioned the PSO as a promising tool for optimizing plasmonic systems in recent years, also in combination with other optimization techniques [33,40,41,42,43]. For this reason, in this work, the optical tuning of a GNA was performed by a customized PSO algorithm implemented in the FDTD method [39,44]. The structure is made of periodically arranged cylindrical holes drilled in a gold layer deposited on a glass substrate. The consequent geometrical model is defined by the cylinder radius and the array periodicity, considering both square and triangular disposition. The PSO dynamic evolution was studied both in terms of convergence and performance through the tuning and efficiency of the GNA optical response.

## 2. Materials and Methods

**Figure 1:**
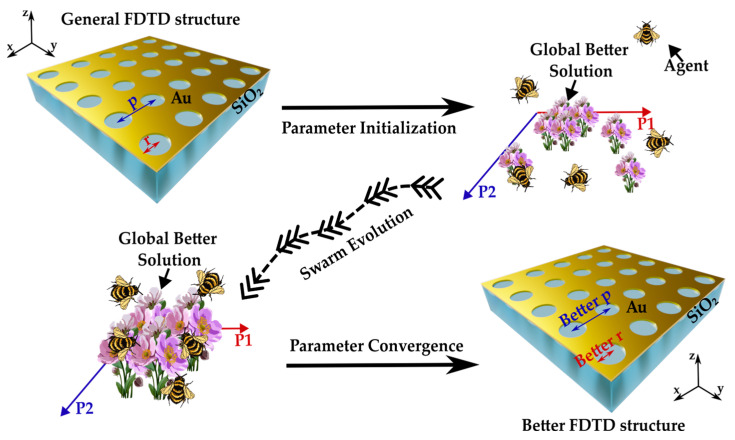
Particle swarm optimization algorithm flow diagram (from upper left to lower right). At the end of the evolutionary process, the algorithm provides a global better solution, i.e., a (Better r, Better p) pair of geometrical parameters corresponding to the FDTD structure with the optimized optical response.

Figure 1 schematically depicts the evolution of the PSO algorithm. The GNA structure is geometrically defined by two values: the pitch (p), accounting for the array periodicity, and the radius (r), accounting for the cylinder radius of the air hole.

Consequently, a properly limited bidimensional parameter space can be defined where the vector of generic coordinates (r,p) is called agent. At this point, the PSO (i) randomly generates a swarm of agents, (ii) builds the corresponding GNA structure inside Ansys Lumerical FDTD [45], and (iii) runs the simulations to extract the optical response of the main localized plasmonic mode sustained by each GNA structure. The final scope of the PSO is to seek for the GNA structure that enables the maximum energy storage inside the localized plasmonic mode in the interval (770 ± 25) nm. The tuning wavelength was chosen as 770 nm for consistency with the sensing device used in [18]. For our sensing purposes, we target the main localized plasmonic mode ability in detecting refractive index variations close to the surface, whose fingerprint is the main minimum in reflectance (R). Practically, this is achieved by calculating through an iterative process the agent’s trajectories (swarm evaluation) identified by stochastic vectors, called velocities (v_r_, v_p_), generators of each agent. A proper fitness function (FF) is defined to scan the parameter space and used at each iteration step to find the best global solution implemented inside a feedback process. In our case, the FF is defined as

FF = 1 − R − T,(1)

where R and T represent the collected reflectance and transmittance spectra evaluated from the corresponding FDTD simulation. Consequently, the FF values identify the evolution of the dynamical system across the iteration steps. Eventually, the algorithm convergence is determined by two independent but mutually necessary requirements: (i) the velocities tend to vector (0,0), meaning that the agents collapsed in the same particular position of the parameter space, and (ii) the FF reaches the highest value. These two conditions are excellent indicators of the PSO capability in converging to the final better FDTD structure.

The FDTD structural model details are reported in Appendix A.1.

## 3. Results

### 3.1. PSO Algorithm Convergence

**Figure 2:**
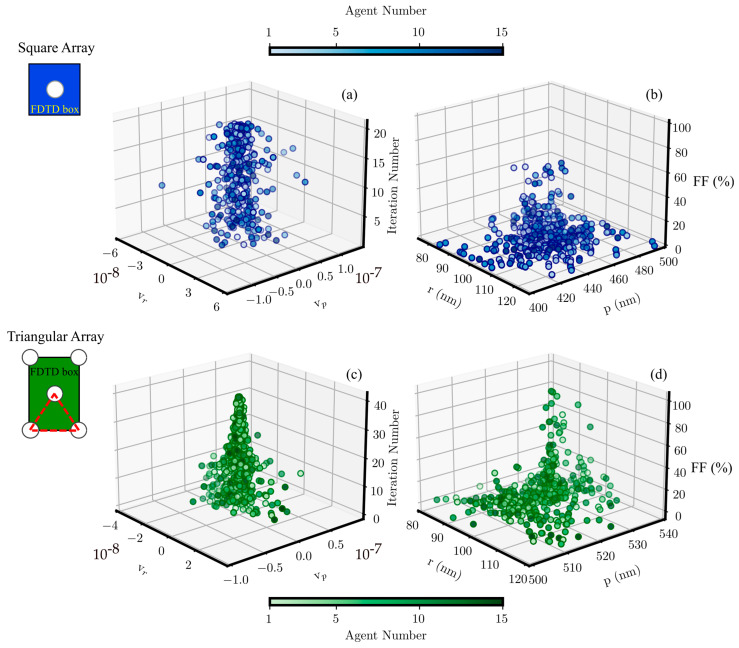
Three-dimensional scatter plots for the square and triangular arrays. Panels (**a**,**c**) show the iteration number against the velocity vectors. Panels (**b**,**d**) show the FF values against the agents.

Figure 2 displays the PSO convergence analysis in the particular case of gold thickness equal to 100 nm, for both square and triangular arrays. The 3D scatter plot of the velocities against the iteration number together with the scatter plot of the FF in the parameter space are shown. In particular, the PSO convergence was studied considering four different gold thicknesses: 100 nm, 80 nm, 60 nm, and 40 nm. The 100 nm value is the standard in the sensing device used in [18]. On the other hand, the lower thickness of 40 nm was chosen as the closest to the skin depth of the material to guarantee simulation reliability. The inferior and superior limits of (r,p) were properly customized for each gold thickness. In consideration of the convergence as defined above, the number of iterations required to obtain the best balance between all the parameters playing in the evolution was observed to be 20 for the square array and 40 for the triangular array. In both cases, the number of agents was fixed at 15 by algorithm stability considerations in the literature [19].

For both arrays, the velocity vectors are scattered around the (0,0) point. In the square array case, the scattering amplitude of the velocity vectors is larger and almost constant, while for the triangular one, it is smaller and tends to diminish along the algorithm evolution. The possible peculiar combination between the computational elements, i.e., FDTD box, source, and metasurface, can result in a symmetry able to be defined on a common basis. In fact, in the case of the square array, these three objects share the same basis, whereas for the triangular array, this set does not consist of an orthonormal combination shared with the FDTD box and source. This combination markedly influences the triangular array case, where the evolution is clearly boosting towards higher FF, stochastically selecting agents step by step closer to the solution. The FF scatter plot in the parameter space is reported in panels (b) and (c) of Figure 2 for the square and triangular arrays, respectively.

### 3.2. Fine-Tuning Procedure

**Figure 3:**
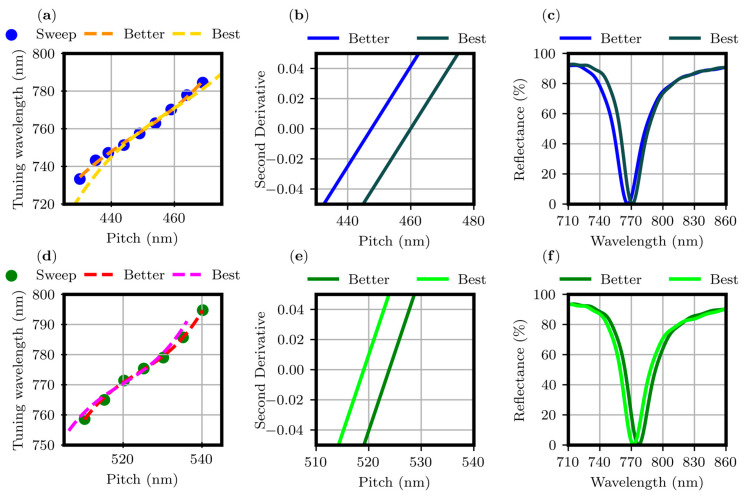
Main steps of the fine-tuning procedure for the square (first row) and triangular (second row) arrays. Tuning wavelengths of the R minimum plotted versus the array pitch in panels (**a**,**d**). Second derivative of the fit (panels (**b**,**e**)). Reflectance spectra of the better and best FDTD structures (panels (**c**,**f**)).

Within the number of iterations, the PSO algorithm in combination with the FDTD method, provides a (Better r, Better p) pair and the better FF value that the optimization routine can provide. Due to the numerical nature of the process, the optical response of the better FDTD structure shows a mismatch in the tuning wavelength within the tuning interval. To save computational time and compensate for this difference, it was necessary to go beyond the PSO limits. To achieve this, an analytical fine-tuning procedure was developed. Operatively, the *p* values were swept in steps of 5 nm in both directions, starting from Better p. For example, the main steps of the fine-tuning procedure for the gold thickness value of 100 nm are detailed in Figure 3.

The tuning wavelengths corresponding to the p values of the sweep are reported in panels (a) and (d) of Figure 3 for the square and triangular arrays, respectively. Considering the disposition of the points, within the tuning interval, the data were fitted by a third-order polynomial curve with χ^2^ values close to 1 (0.995 and 0.997 for the square and triangular array, respectively). The inflection point of the cubic curve systematically corresponds to the (Better r, Better p) pair found by the PSO algorithm. Considering the goodness of the χ^2^ values, the easiest way to shift the fit to properly tune the optical response while preserving the FF value is to apply a second derivative analysis, as visible in panels (b) and (e) of Figure 3, keeping the ratio between the Better r and Better p constant considering a linear approximation for small variations. Eventually, a retuned FDTD structure is generated, and the optical response consistency is verified. Panels (c) and (f) show the R spectra for the retuned FDTD structure where (Better r, Better p) is now identified as (Best r, Best p). With the developed procedure, it is possible to rigorously tune at the desired wavelength the R minimum of the FDTD structure resulting from the optimization routine. The (Better r, Better p) and the (Best r, Best p) values are reported in Table 1 and Table 2 for the square and triangular arrays, respectively.

## 4. Discussion

To test the reliability of the fine-tuning procedure, we decided to perform a comparison using an alternative computational package, specifically EMUstack, based on an open-source code [46]. EMUstack combines Bloch mode expansion in a scattering matrix-like formalism and finite-element method (FEM). In this respect, it is considered more rigorous than FDTD. On the other hand, FDTD allows one to better mimic the experimental configuration for the targeted application and thus improve the implementation and the evaluation of real structures. Both methods, even if completely different, have proven to perform well in the computation of the optical responses of plasmonic systems.

**Figure 4:**
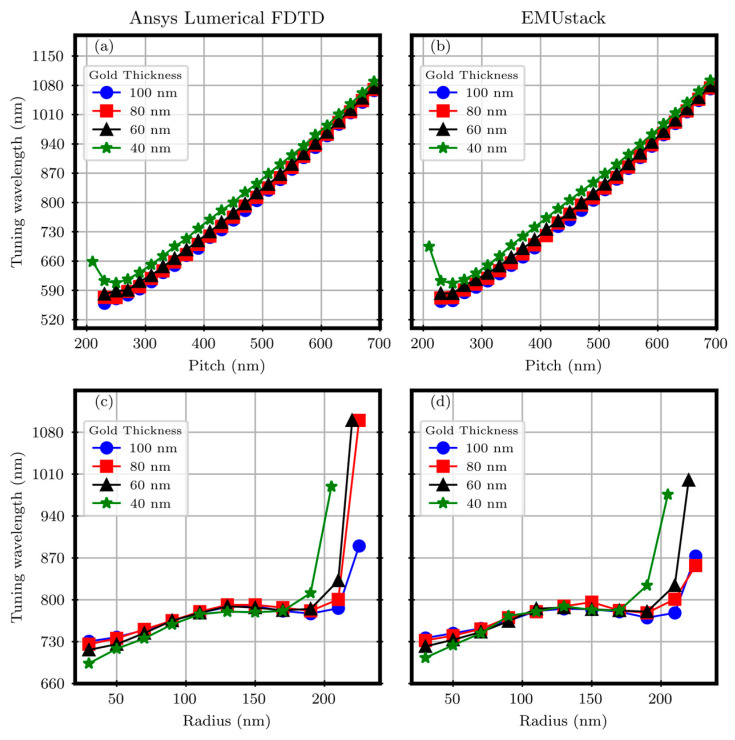
Sweep on the array pitch, panels (**a**,**b**), and cylinder hole radius, panels (**c**,**d**), against the tuning wavelength for the square array.

**Figure 5:**
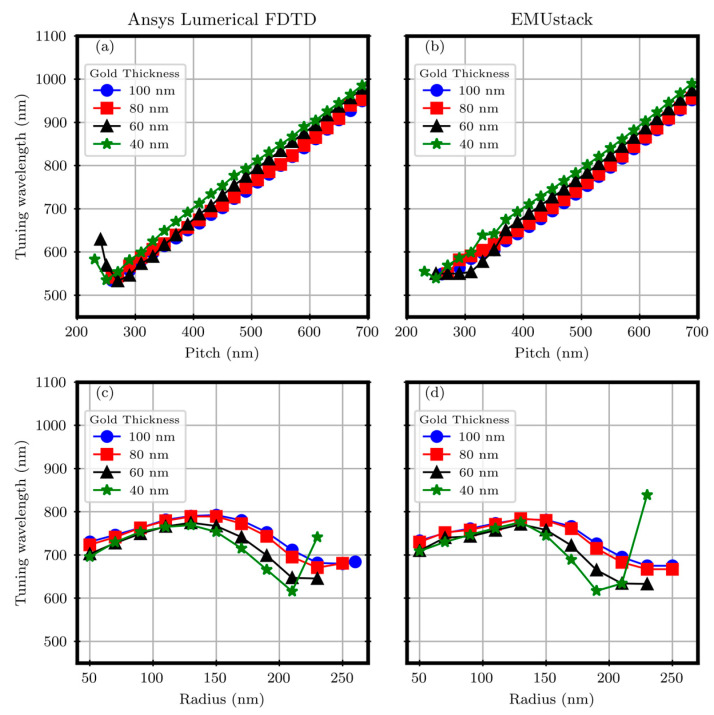
Sweep on the array pitch, panels (**a**,**b**), and cylinder hole radius, panels (**c**,**d**), against the tuning wavelength for the triangular array.

Operatively, starting from the (Best r, Best p) pair, two separate sweeps on p and r were performed with both EMUstack and Ansys Lumerical FDTD [45]. Operatively, starting from Best r (reported in Table 1 and Table 2), the p values were swept below it to the limiting case of the p equal to the hole cylinder diameter and above it, until the R minimum spectral position fell within the range useful for Si-based detectors (400 ÷ 1100) nm. However, starting from Best p, the r was swept from the laser lithography resolution limit (30 nm) to the point where the diameter approached the value of the array pitch. The simulation parameters are reported in Appendix A.1. The results for the square and triangular arrays are reported in Figure 4 and Figure 5, respectively.

Regardless of the gold thicknesses, for both the sweep results on r and p, good consistency between FDTD and EMUstack is observed with discrepancies in the R minimum spectral positions of the order of 10 nm. Regarding the p dependence, as visible in panels (a) and (b) of Figure 4, the data are in agreement within the whole spanned p interval, from 200 nm to 700 nm. In the region below 250 nm, the cylinder hole diameter becomes comparable with p, and therefore, the computational method is no longer accurate, especially for EMUstack, due to the too-demanding computational requirements. A nice consistency can be observed in the r dependence too, for r values from 50 nm to 170 nm, with discrepancies of the order of 15 nm, as visible in panels (c) and (d) in Figure 4. For values outside the interval, either the computation is no more trustable or the r values are too low for real sensing applications and for competitive nanofabrication techniques.

Also, for the triangular array, the sweeps show good agreement between the two computational methods as visible in Figure 5. Nevertheless, the discrepancies are slightly higher, up to 30 nm.

### Plasmonic Mode Dispersion

The high flexibility of Ansys Lumerical FDTD software (release 2023 R2.3, v8.30.3578) allows for further analysis of the physics behind GNAs. In particular, we are interested in studying the dispersion of the GNA optical modes. Energy dispersion is an intrinsic property of SPPs which can be analytically accounted for from the dielectric function [1]. When a periodic array is considered, the folding effect at the symmetry points must be also evaluated and a description in terms of the Brillouin zone has to be used for both SPPs as well as for the pure electromagnetic modes (Wood’s anomalies) [13,16,47,48,49]. Moreover, the presence of holes with a finite size is driving the opening of band gaps at the symmetry points as well as the appearance of almost dispersionless localized modes. Experimentally, it is possible to follow the plasmonic modes’ dispersion features by variable-angle reflectance (R) or transmittance (T) spectra [21,29,50].

At a computational level, the energy dispersion curves of photonic modes with respect to the in-plane (the x-y plane in Figure 1) wavevector component are carried out using well-established techniques [51,52,53] which are typically applicable to dielectric photonic crystals. Due to the inherently lossy nature of GNAs, FDTD simulations provide an effective tool for this investigation. For this reason, we developed a customized script to calculate the plasmonic mode dispersion exploiting Ansys Lumerical FDTD [54].

**Figure 6:**
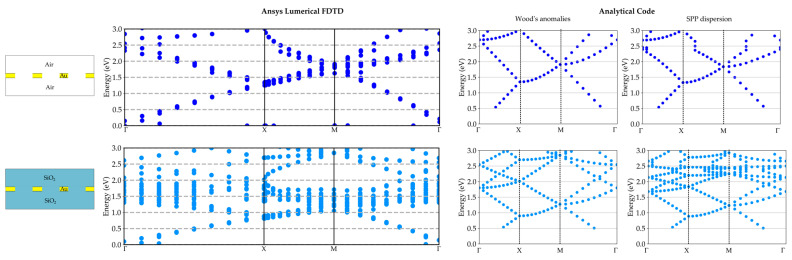
Plasmonic mode dispersion in adimensional units for the square array. First column: results for the suspended metasurface in air and embedded in SiO_2_. Gold thickness set to 100 nm, Best p equal to 459 nm, and Best r equal to 95 nm. On the right: Wood’s anomalies and SPP dispersion with folding.

**Figure 7:**
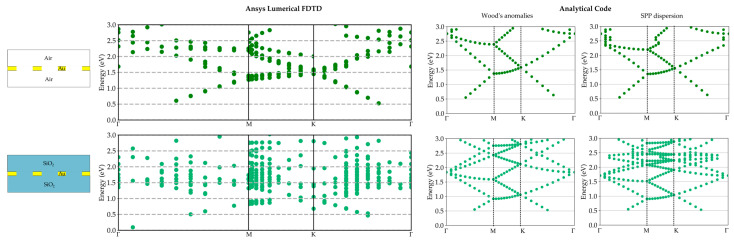
Plasmonic mode dispersion in adimensional units for the triangular array. First column: results for the suspended metasurface in air and embedded in SiO_2_. Gold thickness set to 100 nm, Best p equal to 519 nm, and Best r equal to 97 nm. On the right: Wood’s anomalies and SPP dispersion with folding.

Firstly, we considered the energy dispersion curves for the suspended gold metasurface in air and embedded in SiO_2_. The results are shown in the left columns of Figure 6 and Figure 7 for the case of gold thickness of 100 nm. Also in this case, we wanted to verify the validity of our customized script by comparing it with the analytical code reported in [55]. This code computes the folding of the Wood’s anomalies (light lines) and the SPP dispersion inside the Brillouin zone.

The outcomes are reported in the second and third columns of Figure 6 and Figure 7 for the square and triangular array, respectively. The dispersion relationships are reported in Appendix A.3.

For both array dispositions, the main dispersion features can be clearly distinguished for both methods. Thus, FDTD can be used and trusted for the computation of plasmonic mode dispersion curves in GNAs. Moreover, analytical codes are needed to filter out the computational artifacts created by the FDTD and as a guide to improve and refine the energy dispersion curves. Nevertheless, it has to be pointed out that the FDTD results account for the real array and also the plasmonic band gap opening resulting from the presence of the holes [15,16,49], which are not considered in the analytical approach.

**Figure 8:**
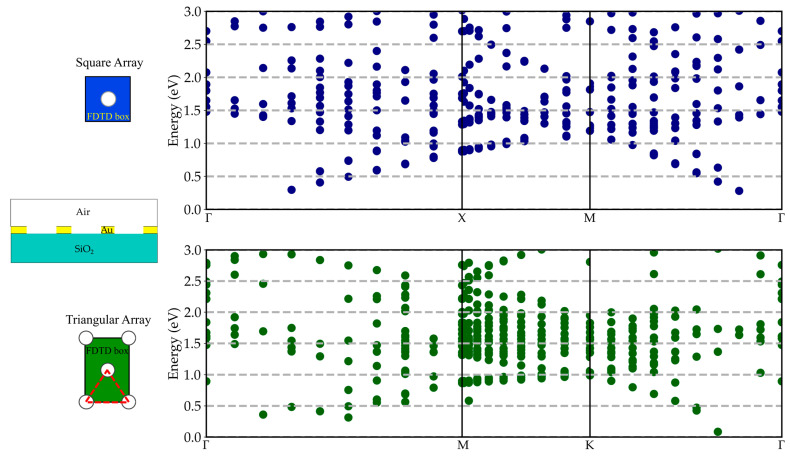
Plasmonic mode dispersion in adimensional units for the square and triangular array considering the actual GNA structure with a gold thickness of 100 nm, while pitch and radius are equal to 459 nm and 95 nm, respectively, for the square array and equal to 519 nm and 97 nm for the triangular array.

For completeness, the energy dispersion curves were calculated for the actual GNA structure, and the results are shown in Figure 8. The complicated nature of the plasmonic mode dispersion is evident: both Woods’ anomalies and SPPs at the Au/air and Au/SiO_2_ interfaces are present, generating a complex behavior due to their mutual interaction [28]. Nevertheless, the results suggest the possibility of developing a new, reliable approach to study the outcomes of the whole optimization procedure in terms of more complex GNA physical properties.

## 5. Conclusions

In this paper, we implemented a customized PSO algorithm in Ansys Lumerical FDTD. The PSO algorithm was able to successfully provide tuned GNA structures with a specific optical response. Consequently, we studied the convergence behavior for a set of optimized parameters (Better r, Better p) and FF. To verify the reliability of the results, we compared the optimization routine based on the FDTD method with a solution provided by a code based on the scattering matrix formalism. The accordance between the results suggests the validity of our approach. In consideration of these outcomes, we decided to test the FDTD capability also in calculating the plasmonic mode dispersion. Again, the outcomes were successfully compared with a specific code based on a direct analytical formalism. Therefore, FDTD proves to be a versatile and reliable method to perform a comprehensive study of GNAs. Experimental validation has already been planned of the optimized GNAs giving a suitable alternative to the actual metasurfaces currently in use. Furthermore, validation tests on their sensing capabilities will be actuated and full quantum method-based simulations [56,57,58] will be exploited to further probe the plasmonic properties.

## Figures and Tables

**Table 1:** (Better r, Better p) and (Best r, Best p) values resulting from the optimization procedure for the square array.

Gold Thickness (nm)	Better r (nm)	Better p (nm)	Best r (nm)	Best p (nm)
100	97	454	95	459
80	99	469	97	455
60	100	442	98	446
40	102	425	101	420

**Table 2:** (Better r, Better p) and (Best r, Best p) values resulting from the optimization procedure for the triangular array.

Gold Thickness (nm)	Better r (nm)	Better p (nm)	Best r (nm)	Best p (nm)
100	98	525	97	519
80	101	532	98	513
60	119	492	117	487
40	106	466	105	463

## Data Availability

Data are contained within the article.

## Appendix A

### Appendix A.1. FDTD Parameters and Structural Model

For each array disposition (i.e., square or hexagonal), air cylinders embedded in a gold film are placed on a semi-infinite SiO_2_ substrate. The dielectric functions of the gold film are set by the built-in Johnson and Christy data in the material database, while the cylinders and the substrate are modeled as dielectric materials with refractive indices of 1 and 1.5, respectively. A plane wave source linearly polarized along the x direction, was placed in the SiO_2_ substrate impinging on the structure at normal incidence. The spectral interval of the source was set to (500 ÷ 1100) nm.

Symmetric and anti-symmetric boundary conditions of the FDTD box were set along the x and y directions, respectively, while perfectly matched layers were set along the z direction. An auto-non-uniform mesh accuracy of 5 was selected after convergence testing.

A conformal variant mesh override refinement of type 2 was selected with a 4 nm mesh step along all the directions. Both R and T spectra were recorded by two frequency domain field and power monitors placed 130 nm below the Au/SiO_2_ and at 150 nm from the gold/air interface, respectively.

The simulations were run on a liquid-cooled Intel^®^ Core 12th generation i7-12700K (12 core) and 64 GB of DDR5-RAM and a liquid-cooled Intel^®^ Core 12th generation i9-12900K (16 core) with 128 GB of DDR5-RAM. An average simulation time of about 6 h was required on the i7 for the square array, while about 10 h were required for the hexagonal arrays on the i9.

### Appendix A.2. EMUstack Parameters

In the EMUstack simulations, the SiO_2_ substrate and air were set as dielectrics with refractive index of 1.5 and 1.0, respectively.

The gold dielectric function was modeled with the Johnson and Christy data. The simulations were run within the same spectral range of FDTD by impinging with a plane wave source at normal incidence. For the square array, after convergence testing, the number of plane waves used was set to 4. Concurrently, a 0.15 background mesh finesse was set with first and second mesh refinements equal to 2. For the triangular array, a higher number of plane waves (5) was used while keeping the mesh parameters unchanged. The R spectra were computed, and the minimum coordinates were calculated. Also in this case, the simulations were run on a liquid-cooled Intel^®^ Core 12th generation i7-12700K (12 core) and 64 GB of DDR5-RAM and a liquid-cooled Intel^®^ Core 12th generation i9-12900K (16 core) with 128 GB of DDR5-RAM. An average simulation time of about 20 min was required for the square array, regardless of the hardware, while about 40 min were required for the triangular array.

### Appendix A.3. Analytical Code for Surface Plasmon Polariton Calculation

The code calculates the folding of both the Wood’s anomalies and SPP dispersion inside the Brillouin zone for both square and triangular arrays with the following relationships:

(A1) $${\mathrm{ck}}_{\mathrm{Wood}}=\omega \sqrt{\varepsilon_{d}}\;{\mathrm{ck}}_{\mathrm{SPP}}=\omega \sqrt{\frac{\varepsilon_{gold}\left(E\right)\times \varepsilon_{d}}{\varepsilon_{gold}\left(E\right)+\varepsilon_{d}}}$$

where $\varepsilon_{d}$ and $\varepsilon_{gold}\left(E\right)$ are the dielectric functions for the dielectric (air or SiO_2_) and gold, respectively.
